# Biosystematic, Essential Oil, and Biological Activity Studies on Medicinal Plant *Moluccella* L. (Lamiaceae) Species from Turkey

**DOI:** 10.3390/plants14040542

**Published:** 2025-02-10

**Authors:** Pelin Yilmaz Sancar

**Affiliations:** Department of Biology, Faculty of Science, University of Fırat, Elazığ 23200, Turkey; peyilmaz@firat.edu.tr

**Keywords:** *Moluccella*, systematic, essential oil, antimicrobial, DPPH

## Abstract

This study aims to determine the biosystematic (morphological, anatomical, palynological) characteristics of *Moluccella* (*M. laevis* and *M. spinosa*) species growing in Turkey, the components of their essential oil (EOs), and some of their biological activities (antimicrobial and radical scavenging capacity). In the biosystematic studies, morphological, anatomical, and palynological analyses were performed. The stem, leaf, flower, and seed characteristics of the plants, along with various ecological properties, were examined and the necessary measurements were taken and presented. In the anatomical studies, the root, stem, leaf, and petioles of the species were photographed under a light microscope to determine their anatomical features. Additionally, light microscope and scanning electron microscope studies were conducted to reveal the surface properties of pollen and seeds. The chemical composition of the EOs of the plant samples was analyzed by GC-MS (Gas Chromatography–Mass Spectrometry). The main components of the EOs of the examined species are α-thujene, β-pinene, β-caryophyllene, and 2-pentadecanone. Significant differences have been found in the EO component profiles of *Moluccella laevis* and *Moluccella spinosa*. While a total of 33 components were found in *M. laevis*, 59 components were detected in *M. spinosa*. The EO yield was higher in *M. spinosa* compared to *M. laevis*. The antimicrobial activity was tested using both disc diffusion and the MIC (minimum inhibitory concentration) methods. The results showed that the methanolic extracts prepared from the aerial parts of the plant samples exhibited varying levels of antimicrobial and antifungal activity against the tested microorganisms. The antioxidant capacity of the methanolic extract was measured by DPPH (2,2-diphenyl-1-picrylhydrazyl) radical scavenging capacity. The DPPH radical scavenging capacity increased with the increasing concentrations of both plant extracts. Antimicrobial capacity was higher in *M. spinosa*, while radical scavenging capacity was higher in *M. laevis*. As a result of the obtained data, although the studied species share similar features, they exhibit significant differences in terms of morphological, anatomical, and palynological characteristics. The antimicrobial and radical scavenging capacities of the plants were noteworthy. The data obtained from this study, which are being presented for the first time in the literature, provide a valuable resource for researchers working on this genus.

## 1. Introduction

The Lamiaceae family is a flowering plant family that mostly includes fragrant, annual, or perennial herbaceous plants, and rarely shrubs. This cosmopolitan family contains approximately 250 genera and between 6900 and 7200 species [1]. Members of this family are rich in volatile and aromatic oils, which makes them useful in pharmacology and the perfume industry. Essential oils (EOs) are obtained from these species, which are used as spices or grown as ornamental plants.

*Moluccella* L., a genus belonging to the Lamiaceae family, consists of annual or short-lived perennial plants native to Central and Southwest Asia and the Mediterranean. These plants are characterized by long, upright stems, 1 m or longer branches, serrated leaves, and small, white, fragrant flowers. They are typically recognized for blooming in mid-summer and autumn. The genus, which has only eight species worldwide, is represented in Turkey by two species that grow in the Eastern Anatolia and Mediterranean regions. The most popular species, widely cultivated as an ornamental plant, is *Moluccella laevis* L. (Syria, Turkey, Ukraine, etc.), which is commonly called the “Bell Flower” due to its green colour and bell-shaped leaves. The flowers are densely packed along each flower head, from top to bottom. The small stem thorns are sharp. These plants perform best in cool summer climates. On the other hand, *M. spinosa* L. grows only in the Mediterranean region of Turkey and has a narrower distribution and a slightly larger, spiny calyx compared to *M. laevis*. It is a rare plant, mostly found in North Africa.

There are a few studies in the literature regarding *Moluccella* species. The cytotoxic activity of extracts of *M. laevis* prepared with various solvents has been tested, and it has been reported that the aqueous extracts showed strong cytotoxic effects against Caco-2 (human colon cancer), while the ethyl acetate (EtOAc) extract exhibited potent cytotoxic activity against the MCF-7 (human breast cancer) cell line [2]. In another study, the biological activities of extracts obtained from *M. laevis* using different solvents were investigated, and it was found to exhibit strong anti-inflammatory, antipyretic, and analgesic activities [3]. These activities were attributed to the high levels of phenolic compounds contained in the plant, suggesting that it could play a supportive role in the development of new drugs in the pharmaceutical field [3]. The strong inhibitory activity of some compounds obtained from *M. aucheri* has suggested that this plant could be a good candidate for the treatment of Alzheimer’s disease [4]. The EOs extracted from the aerial parts of *M. spinosa* have been shown to possess effective antibacterial and antifungal activity against microorganisms that infest historical textiles [5]. In another study, the antioxidant and antimicrobial activities of *M. spinosa* were examined, and both aqueous and methanolic extracts were found to have strong antioxidant activity [6]. However, studies on the biological activity and particularly the systematic characteristics of *Moluccella* species from Turkey and nearby countries are quite limited.

Therefore, this study aims to determine the detailed systematic characteristics of *Moluccella* species distributed in Turkey, along with the EOs components, antimicrobial properties, and radical scavenging capacities of the above-ground parts of the plant samples. It is believed that this study will fill the existing gap in the literature and provide a valuable resource for future research intended to be conducted using these plants.

The data obtained will help in standardizing the identification process of the plants by revealing the similarities and differences both within and between species from a broad perspective, ensuring accurate identification. Furthermore, by presenting these data for the first time, this study will provide additional resources for future research on this topic. To the best of our knowledge, this study is the first report that evaluates the systematic (morphological, anatomical, palynological) characteristics, EO components, and biological (antimicrobial and radical scavenging capacity) activities of *M. laevis* and *M. spinosa* species together.

## 2. Materials and Methods

### 2.1. Plant Materials

Plant materials were collected from their natural habitat in April–July 2023 and brought to the Herbarium of Firat University (FUH) for identification. Some of the collected plants were preserved as herbarium specimens for morphological studies, while others were dried in the shade for EO, antimicrobial, and radical scavenging capacity studies. Another portion was preserved in 70% ethanol for anatomical examinations. The mature flowering parts were collected for palynological studies, while the seeds were stored for micromorphological studies. The detailed locality information is provided in Table 1.

### 2.2. Morphological Observations

Morphological studies consisted of field observations of the samples and macroscopic and microscopic examinations of the samples that were transformed into herbarium specimens in the laboratory. To determine the minimum and maximum values of the examined characteristics, 10 samples were collected from each location and measurements were taken. Measurements of small structures were performed under a stereo microscope (Leica-L2, Germany), while measurements of macroscopic structures were made with the naked eye using a ruler. In addition, the micromorphological characteristics of the seeds were determined using SEM (FIB-SEM, Thermo Fisher Scientific—Waltham, MA, USA). The results are summarized in Table 2.

### 2.3. Anatomical Studies

**Figure 1:**
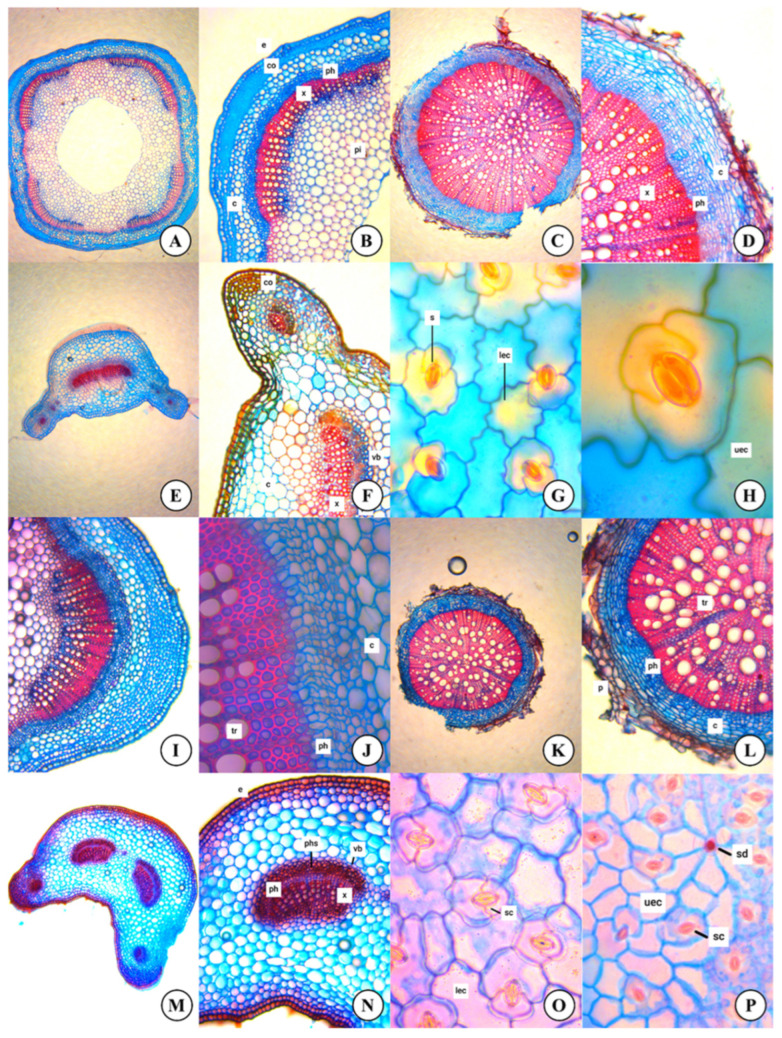
Light microscopy: stem, root, petiole, and leaf cross-sections of the *Moluccella* species ((**A**,**B**) *M. laevis* stem, (**C**,**D**) *M. laevis* root, (**E**,**F**) *M. laevis* petiole, (**G**,**H**) *M. laevis* leaf, (**I**,**J**) *M. spinosa* stem, (**K**,**L**) *M. spinosa* root, (**M**,**N**) *M. spinosa* petiole, and (**O**,**P**) *M. spinosa* leaf. uec: upper epidermis cell; lec: lower epidermis cell; s-sc: stoma cell; e: epidermis cell; tr: trachea; p: periderm; c: cortex; co: collenchyma; ph: phloem; x: xylem; sd: secretory duct; vb: vascular bundle; phs: phloem sclerenchyma; pi: pith region).

Anatomical studies were conducted on plant parts that were preserved in 70% ethanol during the fieldwork. In this study, detailed anatomical structures of the root, stem, and leaf were examined by preparing sections and staining them using the counterstaining method Safranin–Alcian Blue (2:3), and images were captured under a light microscope (ZEISS-Primostar-3, Germany). The results are shown in Figure 1 and Table 3. As a result of the studies, anatomical features with taxonomic importance that allow the distinction of taxa were identified [7].

### 2.4. Palynological Evaluation

**Figure 2:**
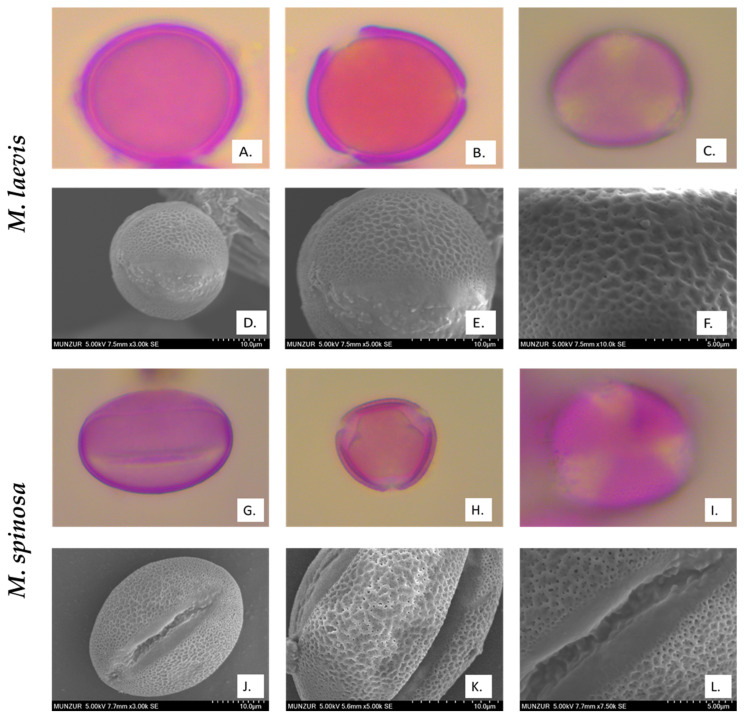
LM (at 100 magnification) and SEM microphotographs. ((**A**–**F**) *M. laevis*, (**G**–**L**) *M. spinosa*, (**A**,**G**) equatorial view, and (**B**,**C**,**H**,**I**): polar view).

Pollen obtained from dried flower samples was examined under a light microscope using the Wodehouse method [8,9,10]. In addition, the pollen was analyzed using SEM (FIB-SEM, Thermo Fisher Scientific) to determine the surface micromorphological characteristics. A total of 4–5 slides were prepared for each sample. The polar axis (P), equatorial axis (E), colpus length (Clg), and colpus width (Clt) were measured from at least 20 fully developed pollen grains using a light microscope (ZEISS Primostar-3, Germany). These measurements are reported in Table 4, and the images are shown in Figure 2. The pollen was identified using the terminology of Faegri and Iversen [11].

### 2.5. EO Studies

For this stage of the study, a large number of plant samples were collected and dried in the shade. The isolation of EOs was performed by the classic hydrodistillation method from dry plant parts (~200 g) placed in a Clevenger apparatus. The working principle of the hydrodistillation method involves boiling dry plant parts placed in a Clevenger apparatus and the separation of the water–oil mixture as it passes through the cold column [12]. The obtained EO was analyzed using GC and GC-MS. The system used (Shimadzu-QP2020, Kyoto, Japan) was equipped with an RXI-5MS (30 m × 0.25 mm × 0.25 µm) capillary column, and helium was used as the carrier gas. According to the working principle of the device, the temperature was set to 40 °C and maintained for 2 min, then heated to 240 °C at a rate of 3 °C per minute. The injection volume was selected as 1 µL in split mode (3 replicates). The column and analysis conditions were the same as described above with GC-MS. The percentage composition of the EOs was calculated from the GC-FID peak areas without correction factors. The MS results were compared with the Wiley-Nist W9N11 libraries stored in the device’s memory (Table 5).

### 2.6. Antimicrobial Sensitivity

The aerial parts of *M. laevis* and *M. spinosa* plants were dried in the shade and ground into powder. Then, 50 mL of methanol (98.1%) was added to the ground plant (10 g), and it was kept in a shaker (100 rpm) for 24 h. The plant materials were filtered under suitable aseptic conditions, and the obtained extract was concentrated by evaporating the methanol. The concentrate was then stored at 4 °C for further studies. Then, 300 µL (60 mg/µL) plant extracts were injected into 6 mm diameter (Schleicher & Shüll No: 2668, Germany) blank antibiotic paper discs to try the test isolates separately [13].

Bacteria (*Escherichia coli* ATCC 25922, *Pseudomonas aeruginosae* ATCC27853, *Enterococcus faecalis* ATCC 29212, *Staphylococcus aureus* ATCC 29213), yeast (*Candida albicans* RSKK 02007, *Candida glabrata* RSKK 014019, *Candida tropicalis* RSKK 02011), and dermatophyte fungi (*Epidermophyton flococusum* RSKK 14024) species were tested in the current study. The bacterial strains tested were obtained from the Infectious Diseases Clinical Microbiology Laboratory, Medical Park Hospital, Istanbul, Turkey; the yeast and dermatophyte fungi were obtained from the Refik Saydam National Drug Culture Collection.

#### 2.6.1. Antimicrobial Activity According to the Agar Disc Diffusion Method Sensitivity Test

Antimicrobial tests were performed using the agar disc diffusion method. Mueller Hinton Agar (Difco), Yeast Malt Extract Agar (Difco), and Sabouraud Dextrose Agar (Oxoid) were sterilized separately in Erlenmeyer flasks and cooled to 45–50 °C. Bacterial, yeast, and fungal cultures were prepared as described above and inoculated at a concentration of 1% (according to the McFarland standard, 10^6^ cells/mL for bacteria, 10^4^ cells/mL for yeast, and 10^4^ cells/mL for dermatophyte fungi). After mixing, 15 mL of the medium was added to the wells of each sterile Petri dish with a 9 cm diameter, ensuring that the medium was evenly distributed.

The 6 mm diameter discs were impregnated with 500 μL (100 mg/μL) of the plant extracts and placed on the inoculated Mueller Hinton Agar, Malt Extract Agar, and Sabouroud Dextrose Agar, respectively. The 9 cm diameter sterile Petri dishes were kept at 4 °C for 2 h. The inoculated plates were then incubated at 37 ± 0.10 °C for 24 h for bacterial strains and also at 25 ± 0.10 °C for 72 h for yeasts and dermatophyte fungi. The antimicrobial activity was evaluated by measuring the zone of inhibition against the test organisms [14]. Standard antibiotics mikostatin and ampicillin–sulbactam were used as a positive control for the yeasts and bacteria. The discs injected with methanol served as negative controls. Each assay in this experiment was replicated three times. The results are summarized in Table 6.

#### 2.6.2. Antimicrobial Sensitivity According to the Minimal Inhibition Concentrations (MICs)

Minimal inhibitory concentrations (MICs) were detected using the broth dilution assay. The cultures were obtained in Mueller Hinton Broth (Difco). The passages of microorganisms were prepared with 12 h broth cultures and the passages were set at a blur of 0.5 McFarland Standard. The extracts were first rare-filled to the maximum value of 500 µL to be evaluated, and then serial two-fold subtilizations were acquired from 62.5 to 500 µL in microtiter plates including nutrient broth for the bacteria and Sabouraud Dextrose Broth for the yeast and dermatophyte fungi. The MIC values of these extracts against the microorganisms were revealed using the micro-well dilution method [15]. The propagation of microorganisms was determined using a universal microtiter plate reader (BioTek EL×800, USA) at 600 nm with optical density quantity after incubation for 18–24 h at 37 ± 1 °C for the bacteria and 25 ± 0.1 °C at 72 h for the yeast and dermatophyte pathogens. It was defined as the smallest value of that sample for the nominal value of the plant extracts used to prevent the proliferation of microorganisms. This value was the last the last clear tube where microbial growth was not observed (mg/µL). The results are summarized in Table 7.

### 2.7. DPPH Radical Scavenging Assay

The antioxidant activity was evaluated using the DPPH (2,2-diphenyl-1-picrylhydrazyl) free radical scavenging capacity method [16]. The solution was prepared by dissolving the obtained extract in methanol at a concentration of 1000 mg/mL. The prepared solution was diluted four times, and a calibration curve for DPPH was obtained. From the prepared solution, 40 µL was taken, and 160 µL of DPPH solution was added. After thorough mixing, the mixture was sealed and kept in the dark for 30 min. The free radical scavenging ability of the samples was determined by measuring the absorbance at 570 nm using an Elisa microplate reader (Allsheng Amr-100, Hangzhou, China) [17]. The results are summarized in Table 8. Vitamin C and methanol were used as positive and negative controls, respectively. The % inhibition values were calculated using the following formula:

DPPH radical scavenging activity (%) = [(A_0_ − A_1_)/A_0_] × 100

In this equation, A_0_ and A_1_ refer to the absorbance values of the methanol and plant samples, at 570 nm, respectively.

### 2.8. Statistical Analysis

Radical scavenging capacity analyses performed in triplicates were conducted using SPSS 21.0 version. To evaluate the significant relationships between experimental parameters, analysis of variance (ANOVA) was performed, with *p* < 0.01 considered statistically significant.

The statistical comparisons of the plant extracts and the control group (ampicillin–sulbactam, nyctatine) were made with respect to the measurable antimicrobial activity against test microorganisms. SPSS 15.0 software was used for the statistical analysis. The results obtained with the analysis of variance (ANOVA) and least significant difference (LSD) tests were given as mean ± standard error (mean ± SD). *p* < 0.0001, *p* < 0.001, and *p* > 0.05 were used for the differences between the groups. *p* < 0.0001 and *p* < 0.001 were considered highly significant and significant.

## 3. Results

### 3.1. Morphological Results

Although the studied species belong to the same genus, each species has characteristic features that allow them to be easily distinguished from each other. *M. laevis* is notable for its pale green colour. It has a hairless stem and leaves. The plant is characterized by dense foliage when young and with fallen leaves at maturity as well as calyces arranged in a bell shape, stacked on top of one another. It has a circular flower arrangement. Each flower ring has spines underneath. The calyx is pale green and distinctly net-veined. The corolla is bilabiate and hairy in structure, white and sometimes light purple in colour. *M. spinosa* has a dark purple stem and green leaves. As the plant matures, the leaves turn purple. It has a circular flower arrangement. The calyx is green when young and turns purple at maturity. It is characterized by spines at the tips. The corolla is bilabiate and densely hairy. It is pink and light purple in colour.

According to the data obtained from the studies conducted on the herbarium specimens, the stem lengths, leaf measurements and characteristics, calyx and corolla features and measurements, petiole measurements, flowering types, and hairiness of *Moluccella* species are detailed in Table 2.

### 3.2. Anatomical Features

The stem is quadrangular in both species. The epidermis layer surrounding the stem is rectangular in *M. laevis*, while in *M. spinosa* it is oval–rectangular. Beneath the epidermis, *M. laevis* has a hypodermis layer composed of oval cells, whereas *M. spinosa* lacks this layer. The corner collenchyma at the stem’s corner regions consists of 4–5 rows in *M. laevis* and 6–7 rows in *M. spinosa*. Additionally, in some cross-sections of *M. laevis*, 3–5 rows of oval–flattened parenchyma tissue have been observed beneath the corner collenchyma. Both species have single-layered bundle sheath cells surrounding the vascular bundles. In both species, the vascular bundles show a bicollateral arrangement with the xylem positioned radially in both the inner and outer parts. In *M. laevis*, the phloem layer on the outer side of the xylem consists of 4–5 rows of cells, and the phloem layer on the lower side of the xylem consists of 3–5 rows of cells. The xylem covers a large portion of the stem and consists of 6–12 rows of cells. There is a cavity in the middle part of the stem, and the pith is fragmented. The parenchymatic cells forming the pith are oval or circular in shape. In *M. spinosa*, the phloem layer consists of 5–7 rows of cells, while the xylem consists of 6–12 rows of cells. The phloem layer beneath the xylem consists of 2–4 rows of cells. There is also a cavity in the middle part of the stem, and the pith is fragmented. The parenchymatic cells forming the pith are oval or circular in shape.

The periderm layer in the root consists of 1–3 rows of cells in *M. laevis* and 2–5 rows in *M. spinosa*, made up of flattened rectangular-shaped cells. The cortex cells located below are in 6–8 rows in *M. laevis* and 3–6 rows in *M. spinosa*. The phloem in *M. laevis* consists of 5–7 rows of cells, while in *M. spinosa* it is made up of 5–6 rows. In both species, the xylem covers the entire pith region, and the pith rays are quite prominent. In some cross-sections, the tracheid walls have thickened due to lignin accumulation, forming a sclerenchymatic structure.

In the cross-section taken from the middle of the leaf, similar characteristics are observed in both species. The smaller epidermal cells on both the upper and lower surfaces of the leaf are rectangular in shape, while the larger epidermal cells are oval. The palisade parenchyma cells, located adjacent to the adaxial epidermis, are arranged in a single layer and are usually rectangular and regularly arranged. The spongy parenchyma is 2–4 layers thick and lies between the palisade parenchyma and the abaxial epidermis. The cells of the spongy parenchyma are generally oval or irregularly shaped. In the superficial sections taken from both the upper and lower surfaces of the leaf, the epidermal cells on the lower surface are more densely curled. Both the upper and lower surfaces of the leaf contain stomata, with a higher number of stomata found on the lower surface. The stomata are of the diacytic type. The leaf also contains stellate secretory channels, which are accompanied by secretory hairs. These secretory channels and secretory hairs are present in few numbers in *M. laevis*, while they are present in large quantities in *M. spinosa*.

The petiole is sulcate in shape. The cover hairs on the lower epidermis are sparse in *M. laevis*, while they are denser in *M. spinosa*. Both species have a thick cuticle layer over the upper and lower epidermal cells, and the cells are arranged in a single layer. In *M. laevis*, under the middle vein, there are 1–3 rows of collenchyma and 4–7 rows of parenchyma cells below the epidermis. The mesophyll consists of 1–2 layers of palisade parenchyma and 1–3 layers of spongy parenchyma. No hairs were found in the transverse section of the petiole. The petiole contains a single vascular bundle, with the central vascular bundle being large and crescent-shaped. Two smaller crescent-shaped vascular bundles are located at each corner, and these are surrounded by parenchymatic cells. In *M. spinosa*, under the middle vein, there are 1–2 rows of collenchyma and 4–5 rows of parenchyma cells beneath the epidermis. The mesophyll consists of 1–2 layers of palisade parenchyma and 2–3 layers of spongy parenchyma. No hairs were found in the transverse section of the petiole. The petiole contains four vascular bundles. The central vascular bundle is large, and at each corner, there is a small crescent-shaped vascular bundle surrounded by parenchymatic cells. The explanations of anatomical characteristics have been expanded with detailed studies conducted on *Moluccella* species and are presented in Figure 1 and Table 3.

### 3.3. Palynological Properties

All the determined morphological parameters are shown in Table 4 and Figure 2. Measurements and observations were made under light microscopy (LM), and more detailed ornamentations were identified from SEM images. The pollen grains of the two *Moluccella* species are tricolpate and observed as isopolar. In *M. laevis*, the pollen grains are spheroidal and radially symmetrical. In addition to the reticulate ornamentations, the colpus edges are scabrate. The polar axis (P) ranges from 2.59 μm to 2.99 μm, while the equatorial axis (E) ranges from 2.59 μm to 3.02 μm. The colpus length (Clg) ranges from 2.42 μm to 2.53 μm, and the colpus width (Clt) ranges from 0.55 μm to 0.62 μm.

In *M. spinosa*, the pollen grains are prolate and radially symmetrical. Reticulate ornamentations are observed. The polar axis (P) ranges from 3.30 μm to 3.94 μm, and the equatorial axis (E) ranges from 2.31 μm to 2.91 μm. The colpus length (Clg) ranges from 3.04 μm to 3.13 μm, and the colpus width (Clt) ranges from 0.25 μm to 0.35 μm.

### 3.4. EOs Results

The EOs of *Molucella* consists of various components. In terms of major components, some of the highlighted substances are α-thujene, β-pinene, β-caryophyllene, and 2-pentadecanone. The most abundant components are isobornyl acetate and nonanoic acid, while other components are scattered throughout the oil. The compositions of the EOs of *Molucella* species are listed in Table 5.

The components demonstrate the diversity and chemical variability of the EOs. α-thujene and β-pinene belong to the monoterpene category and are important primarily due to their aromatic and therapeutic effects [7,12]. Additionally, esters such as isobornyl acetate contribute with a softer scent and are widely used in the cosmetic industry [18]. The distribution of these components highlights the potential of the oil for both industrial and therapeutic applications.

There are significant differences in the component profiles of *Molucella laevis* and *Molucella spinosa*. In *M. laevis*, a total of 33 components are found, with isobornyl acetate (28.01%) being the most dominant component. In *M. spinosa*, the absence of this component is noteworthy, while α-thujene (5.93%), β-pinene (5.64%), and β-caryophyllene (5.65%) are other major components present in *M. laevis*.

Moreover, in *M. spinosa*, 59 components were identified, with nonanoic acid (32.19%) standing out, and its ratio is lower in *M. laevis*. 2-pentadecanone (8.65%) and epilongipinanol (5.19%) are two other major components found in *M. spinosa*. When examining other differences between the two plants, it can be seen that *M. laevis* presents a profile rich in monoterpenes (thujene, pinene) and esters, while *M. spinosa* contains more long-chain acids and alcohols. These differences diversify the potential industrial applications of both plants.

This analysis reveals the multi-chemical structure and richness of *Molucella* EOs. The data obtained indicate that the oil carries significant potential for various industrial and therapeutic uses.

### 3.5. Antimicrobial Results

Antimicrobial susceptibility tests are laboratory procedures conducted to determine which antibiotics microorganisms are sensitive or resistant to. These tests are critical for effectively treating infections.

Antibiotic susceptibility refers to the degree to which bacteria are affected by specific antibiotics. This susceptibility determines the ability of bacteria to survive or reproduce in the presence of antibiotics. Antibiotic susceptibility indicates whether disease-causing microorganisms are affected by certain doses of antibiotics; that is, if a microorganism is sensitive to a particular antibiotic, the antibiotic can either kill the microorganism (microbicidal) or inhibit its reproduction (microbistatic) [19].

Table 6 shows the antimicrobial susceptibilities of the tested microorganisms according to the disc diffusion method using plant extracts soluble in methanol to inhibit microorganism growth. The dose of plant extracts absorbed into the discs is 300 µL. Table 7 shows the antimicrobial susceptibilities according to the minimum inhibitory concentration. The first dilution concentration at which these plant species began to inhibit the growth of pathogenic microorganisms was determined to be 500 μL. The analyses were conducted in four repetitions, and the concentration at which microorganism growth was not inhibited was 90 μL.

According to the disc diffusion method using methanol-prepared *M. spinosa*, antibacterial activity was observed against bacteria, with an inhibition zone of 11 mm for *P. aeruginosa*, 10.6 mm for *S. aureus*, and 12.6 mm for *E. faecalis* (effective; *p* < 0.001; d) (Table 6). The control antibiotic, ampicillin–sulbactam, showed a more effective antibacterial sensitivity with an inhibition zone of 19.3 mm, indicating a higher inhibitory effect (*p* < 0.0001; cd) compared to *M. spinosa*. This difference is due to the varying dosages of the antibiotic and the plant extract.

These plant extracts inhibited the growth of only *C. tropicalis* (9.3–10.3 mm inhibition zone) and *E. floccosum* (effective; *p* < 0.001; d) among the yeast and dermatophyte fungal isolates, while it did not show any antifungal activity against the others (a: *p* > 0.05). Among the standard antibiotics, Mycostatin was more effective in preventing the growth of yeast and dermatophyte fungi.

In addition, discs impregnated with methanol do not show an antimicrobial effect at 50 microliters, but are effective at 100 μL. To confirm the data shown by the methanol-impregnated discs, 100 microliters of methanol was added to the well of the microplate and no microorganism growth was observed (on *P. aeruginosa*, *S. aureus*, *E. faecalis*, *C. troicalis*). This result is compatible with the disc diffusion result.

The MIC value is defined as the last clear tube where microbial growth is not observed. The methanol-prepared *M. spinosa* MIC of 62.5 μL (12.5 mg/μL) is the lowest concentration that inhibited the growth of *P. aeruginosa* and *S. aureus*. In addition, the MIC of these plant extracts on *C. tropicalis* and *E. floccosum* was 250 μL. No antimicrobial effect was observed at lower concentrations, as the growth of other microorganisms was not inhibited at these concentrations (Table 7).

The methanolic extract of *M. laevis* was examined using the disc diffusion test, and it was found to exhibit effective antimicrobial activity against both Gram-negative and Gram-positive bacteria (effective; *p* < 0.001; d). The inhibition zone diameters were as follows: 14.33 mm for *E. coli*, 11.6 mm for *P. aeruginosa*, 12.3 mm for *S. aureus*, and 10.6 mm for *E. faecalis* (Table 6).

For yeast and dermatophytes, *M. laevis* exhibited the same characteristics as *M. spinosa*, inhibiting the growth of *C. tropicalis* and *E. floccosum* (28.3 mm/inhibition zone for *C. tropicalis* and 9.3 mm/inhibition zone for *E. floccosum*) (effective; *p* < 0.001; d), while no antimicrobial effect was observed against the other strains (a: *p* > 0.05).

An important finding from the disc diffusion test results of *M. laevis* and *M. spinosa* extracts is that *E. floccosum* showed more effective inhibition compared to the standard antibiotics, indicating that this extracts can be used as alternative natural antimicrobial agents for skin and nail infections (highly inhibitory effect; *p* < 0.0001; cd) (Table 6).

To confirm the results from the microbial plaque test, when the 500 (100 mg/μL) and 250 (50 mg/μL) μL concentrations of *M. laevis* and *M. spinosa* extracts were applied by spreading on agar against *C. tropicalis* and *E. floccosum*, no colonies were observed, while growth was observed for other microorganisms. The control antibiotics, ampicillin–sulbactam and nystatin, showed very effective antimicrobial sensitivity with inhibition zones ranging from 14.3 to 30 mm. When compared to these data, *M. laevis* exhibited microbistatic properties, while the antibiotics exhibited microbicidal properties.

When the MIC value was examined (Table 7), the lowest concentration that inhibited the growth of *P. aeruginosa* and *E. faecalis* was 62.5 (12.5 mg/μL) μL. This dose did not inhibit the growth of other microorganisms, and no antimicrobial effect was observed.

It was observed that a 100 (20 mg/μL) μL dose of methanol in the microplate prevented the growth of *P. aeruginosa*, *S. aureus*, *E. faecalis*, and *E. coli.* The lowest concentration of plant extracts prepared with methanol, which prevents the proliferation of the mentioned microorganisms, is 62.5 (12.5 mg/μL) μL. The reason why methanol prevents the development of microorganisms in 100 μL of plant extracts at 62.5 μL (12.5 mg/μL) is due to the fact that methanol reveals the active beneficial components in the plant.

This study demonstrates that both plant species, at specific concentrations, possess antibacterial and antidermatophytic properties, and could serve as natural agents that may contribute to the components of anti-inflammatory and wound-healing medications. Additionally, the data obtained reveal the existing antimicrobial susceptibilities of the plant extracts and provide an important reference point for future research.

### 3.6. DPPH Radical Scavenging Results

The inhibition percentages of DPPH radical scavenging activity of methanolic extracts from the above-ground parts of *Moluccella* species at different concentrations have been determined (Table 8). It was observed that the DPPH radical scavenging activity of *M. laevis* was above 50% (56.91 ± 0.06, 81.47 ± 0.53, 84.15 ± 0.59) at concentrations of 500, 750, and 1000 mg/mL, while it was below 50% at other concentrations. In contrast, *M. spinosa* showed DPPH radical scavenging activity above 50% only at the concentrations of 750 and 1000 mg/mL. The DPPH radical scavenging effect of vitamin C, used as a positive control, was found to be 99.9 ± 0.2%, while the DPPH radical scavenging effect of methanol, used as a negative control, was 2.7 ± 0.3%. When comparing the obtained results with the controls, it was determined that the closest antioxidant effect to the positive control was found in the methanol extract of *M. laevis* at concentrations of 750 mg/mL and 1000 mg/mL (81.47 ± 0.53, 84.15 ± 0.59) (Table 8). Additionally, it was observed that *M. spinosa* exhibited lower radical scavenging activity compared to *M. laevis*.

## 4. Discussions

In this study, the systematic characteristics of *M. laevis* and *M. spinosa* species, as well as important biological activity traits such as volatile oil components, antimicrobial activity, and radical scavenging capacities, are evaluated together.

To the best of our knowledge, there has not been a comprehensive systematic study of the *Moluccella* species in the literature. In macromorphological examinations of the *Moluccella* genus, no new characteristics have been discovered beyond those described in the literature. In *Moluccella*, the shape of the calyx in particular is an important characteristic for species identification. It has been observed that the morphological measurement values of *Moluccella* species are largely consistent with the findings in the literature [20], although some deviations in the minimum and maximum limits of the measurement values have been noted. For example, in *M. laevis*, leaf measurements (w/l) range from 18 × 15 to 50 × 35, and the plant height ranges from 10 to 70 cm. There are spiny bracts numbering between 8 and 10 under the flower inflorescence. The seeds are arranged in each calyx after the fruit, with four seeds per calyx. The seed shape is a plump triangular structure. In *M. spinosa*, leaf measurements (w/l) range from 23 × 20 to 43 × 38, and the plant height ranges from 20 to 110 cm. The seeds are also arranged in each calyx after the fruit, with four seeds per calyx. Although the seed shape is quite similar to that of *M. laevis*, it is relatively more elongated and slender.

In anatomical studies, root, stem, petiole, and leaf cross-sections have been examined in detail. Abdately et al. also conducted a detailed anatomical study of *M. laevis* [21]. While current data are largely consistent with the literature, the anatomical study of *M. spinosa* is being introduced to the literature for the first time. The roots of both species exhibit similar characteristics, with the periderm layer being relatively thicker in *M. spinosa*, while the cortex is nearly identical. In both species, the xylem completely covers the central region. The stem is distinctly quadrangular. In *M. laevis*, unlike *M. spinosa*, the hypodermis layer is found just beneath the epidermis. In both species, the presence of corner collenchyma at the corners of the stem is highly characteristic and adds strength to the plant. Both species have vascular bundles of bicollateral type, the central region of the stem is fragmented and the stomata on the leaf are diacytic. Additionally, *M. spinosa* contains dense secretory cells, which are fewer in number in *M. laevis*.

Abdately et al. stated that the pollen of *M. laevis* is spherical in shape and has smooth walls [21]. According to the findings obtained from this study, the pollen grains of *M. laevis* are spherical, 3-colporate, and have a reticulate ornamentation. *M. spinosa*, on the other hand, has prolate pollen that is 3-colporate and exhibits reticulate ornamentation.

As a result of current EO studies, 33 components were detected in *M. laevis* and 59 components were detected in *M. spinosa*. The most recent GC-MS analysis of the EOs from *M. laevis* flowers identified α-pinene, chrysanthenyl acetate, and isobornyl acetate as the main components, while the oil from the plant’s leaves contained isobornyl acetate, 2-methyl-4-butanolide, 1-heptene oxide, and methyl benzoate [2]. In another study, the EO components of *M. laevis* were characterized by GC-MS as α-pinene, pinocarvone, methyl chavicol, and β-caryophyllene [22]. The EO of *M. spinosa* was analyzed by GC-MS, and α-pinene, caryophyllene oxide, and β-caryophyllene were reported as the main components [5]. According to current results, isobornyl acetate (28.01%) is the dominant component in *M. laevis* EOs. Notably, this component was absent in *M. spinosa*. Isobornyl acetate is primarily used as a fragrance in perfumes and cosmetics [18]. Additionally, studies have proven that it shows activity against a range of microorganisms, including bacteria, fungi, and viruses [23]. Other major components found in *M. laevis* include α-thujene (5.93%), β-pinene (5.64%), and β-caryophyllene (5.65%). These components are natural organic compounds classified as monoterpenes [24]. Monoterpenes exhibit various pharmacological activities, such as antimicrobial, antiviral, antispasmodic, antifungal, anticancer, antimalarial, antioxidant, and anti-inflammatory effects [25,26,27,28].

One of the prominent components of *M. spinosa* EOs is nonanoic acid (32.19%). The concentration of this compound is lower in *M. laevis*. Nonanoic acid is a straight-chain saturated fatty acid [29]. It has antifungal properties and is commonly used as a herbicide to control weeds [30]. Nonanoic acid inhibits mycelial growth and spore germination in plant pathogenic fungi *M. roreri* and *C. perniciosa* in a concentration-dependent manner [31]. Formulations containing nonanoic acid are used for both indoor and outdoor weed control and as cleansing and emulsifying agents in cosmetics [32]. Other major components found in *M. spinosa* include 2-pentadecanone (8.65%) and epilongipinanol (5.19%). 2-Pentadecanone belongs to a class of organic compounds known as ketones. It is widely used as a lubricant and softener in cosmetics and personal care products to give products a smooth touch. As an important organic solvent, it is commonly used in the production of coatings, inks, and adhesives [33].

Recent studies revealing the antimicrobial properties of plants are quite promising in terms of discovering new plant-based medicinal raw materials. The use of plant-based products in the treatment of diseases can help prevent potential health problems. For this reason, in recent years, studies on the chemical components of various plant sources used as antimicrobial and anticancer agents have increased [34]. Secondary metabolites produced by plants are generally responsible for the biological properties of plant species used worldwide [35,36]. Alkaloids, tannins, flavonoids, and phenolic compounds found in plants are therapeutic for human health [37,38]. In the literature, there are a limited number of antimicrobial studies conducted using *M. laevis* and *M. spinosa* species. In one study, the biological activities of extracts obtained from *M. laevis* using different solvents were investigated, and it was found to show strong anti-inflammatory, antipyretic, and analgesic activities [3]. Some compounds obtained from *M. aucheri* were shown to have strong inhibitory activity, suggesting that this plant could be a good candidate for the treatment of Alzheimer’s disease [4]. The EO obtained from the aerial parts of *M. spinosa* was found to exhibit effective antibacterial and antifungal activity against microorganisms infesting historical textiles [5]. In another study, the antioxidant and antimicrobial activities of *M. spinosa* were investigated, and it was stated that both the aqueous and methanolic extracts had strong antioxidant activity [6]. According to the current results, the methanolic extract of *M. laevis* showed antibacterial sensitivity against *E. coli*, *P. aeruginosa*, *S. aureus* and *E. faecalis*. However, this plant extract did not show any antifungal sensitivity on other microorganisms, only inhibiting the growth of *C. tropicalis* and *E. floccosum* among yeast and dermatophyte fungal isolates.

Similarly, the methanolic extract of *M. spinosa* showed antibacterial sensitivity against *P. aeruginosa*, *S. aureus*, and *E. faecalis*, while it inhibited the growth of *C. tropicalis* and *E. floccosum* among yeast and dermatophyte fungus isolates. However, it did not show any antifungal sensitivity against the other microorganisms studied.

The fact that both plant extracts are more effective than standard antibiotics against *E. floccosum* suggests that these plants could be alternative natural antimicrobial agents that can be used for skin and nail infections.

The antioxidant activity analysis based on the DPPH free radical scavenging capacity of *M. laevis* and *M. spinosa* is also quite significant. As the plant extract concentrations increase, high antioxidant activity is observed. Similarly, a study conducted with *M. aucheri* emphasized the strong antioxidant properties of the plant, particularly its ethanol extract [4]. It has been determined that the DPPH free radical scavenging capacity of *M. laevis* is stronger compared to *M. spinosa.*

## 5. Conclusions

Although there are several studies related to the systematic characteristics of *M. laevis*, the systematic features of *M. spinosa* taxa have been introduced to the literature for the first time with this study. The morphological, anatomical, and palynological information obtained from this research will serve as a valuable resource for researchers studying *Moluccella* and will facilitate the biosystematic analyses of these species.

Many members of the *Lamiaceae* family are frequently found in the medicine, pharmacy, food and spice, cosmetics, and perfume industries due to their high EO content. The results of biological studies, along with the findings from the current research, are very promising in terms of revealing the therapeutic potential of the genus. As a result, the methanolic extracts of *M. laevis* and *M. spinosa* have promising potential, and further analytical and clinical studies are required to isolate the active molecules from these plants and to evaluate their pharmacological effects clinically. This study is the first to conduct biosystematic and biological activity research on *Moluccella* species with a natural distribution in Turkey. The obtained data will contribute to new drug development and design research in the field of pharmacology and will form the basis for the creation of larger-scale projects.

## Figures and Tables

**Table 1:** The locality information of the studied *Moluccella* species.

Species	Locality	Collection Date and Collector Number
*M. laevis*	B7, Elazig; Isikyolu village, roadsides and gardens, 1182 m. Long: 39° 2′37.312″ E Lat: 38° 30′55.825″ N	25 July 2023 P. Yılmaz Sancar 3501
*M. spinosa*	C5, Mersin; Silifke, Atakent neighbourhood, Merdivensay locality, under Ceratonia siliqua, 82 m. Long: 34° 4′41.515″ E Lat: 36° 25′52.426″ N	7 May 2023 P. Yılmaz Sancar 3521

**Table 2:** Morphological and morphometrical characteristics of *Moluccella* species.

	*M. laevis*	*M. spinosa*
**Life cycle**	Annual	Annual or short-lived perennial
**Stems size (cm) and colour**	10–70 cm, initially green and straw-coloured when mature	20–110 cm, initially green and purple when mature
**Hairs of stem**	Glabrous	Glabrous
**Leaf shapes**	Orbicular–ovate, palmate–crenate or incised, longly petiolate	Orbicular–ovate, coarsely palmate lobed or serrate, shortly petiolate
**Width of the leaf (mm)**	18–40	23–43
**Length of leaf (mm)**	15–35	20–38
**Bracteoles (mm)**	Spiny, 5–11	Spiny, 7–15
**Petiole (mm)**	5–30	10–19
**Inflorescens**	Verticillate	Verticillate
**Flowers**	6–12 flowers	8–10 flowers
**Calyx**	Pale green with whitish reticulate veins. Limb greaty expanded, membranous, 20–40 mm across, with 5 mucros, not spiny, abscissing in fruit, with nutlets.	Green-burgundy, reticulate veins. Narrow limb structure, rigid, 15–30 mm across, with 8 mucros, rigid and spiny, ersisting in fruit, with nutlets
**Corolla**	c. 20–25 mm; upper lip entire, shortly hairy. Purplish-pink or mauvish-white	c. 20–40 mm, upper lip broadly emarginate or bifid, villous. Pale pinkish-violet or white.
**Fl.**	6–8. Fallow and cornfields, waste ground, vineyards, eroded bank.	4–6. Stony ground (on lime-stone).
**Alt.**	c. 100–1300 m	c. 0–600 m
**Seed**	4 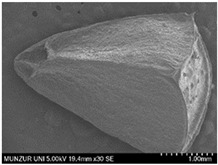	4 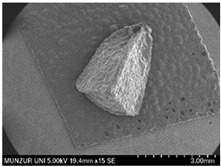

**Table 3:** Comparative anatomical measurements of investigated *Moluccella* species (μm).

	*M. laevis*	*M. spinosa*
**Stem**		
**Collenchyma layers**	4–5	6–7
**Phloem layers (inner)**	4–5	5–7
**Xylem layers**	6–12	6–12
**Phloem layers**	3–5	2–4
**Root**		
**Periderm layers**	1–3	2–5
**Cortex layers**	6–8	3–6
**Sclerenchyma layers**	3–5	3–5
**Pith region**	10–18	12–20
**Petiole**		
**Petiole shape**	sulcat	sulcat
**Collenchyma cell layers**	1–3	1–2
**Parenchyma cell layers**	4–7	4–5

**Table 4:** Qualitative and quantitative micromorphological pollen features via microscopic measurements (μm).

Taxa	Shape	Type and Number of Apertures	Exine Ornamentation	P	E	P/E Ratio	Clg	Clt
***M. laevis***	Spheroidal	3-colporate	Reticulate	2.80	2.82	0.99	2.47	0.58
***M. spinosa***	Prolate	3-colporate	Reticulate	3.91	2.57	1.57	3.08	0.3

**Table 5:** The chemical composition of EOs in the *Moluccella* species.

No	Components	RT	IM	*M. laevis* %	*M. spinosa* %
1.	α-Thujene	9.89	RI, MS	**5.93**	-
2.	α-Pinene	11.49	RI, MS	2.85	-
3.	Sabinone	12.45	RI, MS	2.87	-
4.	β-Pinene	13.52	RI, MS	**5.64**	-
5.	*p*-Cymene	14.00	RI, MS	1.55	-
6.	α-Phellandrene	14.92	RI, MS	1.13	-
7.	Limonene	16.18	RI, MS	2.00	-
8.	2-Pentyl furan	20.01	RI, MS	0.87	-
9.	Alloocimene	20.76	RI, MS	1.83	-
10.	Δ-Carene	21.54	RI, MS	3.21	-
11.	1.8-Cineole	22.01	RI, MS	0.74	-
12.	*Trans*-Camphor	24.87	RI, MS	0.22	-
13.	α-Camphoneal	25.98	RI, MS	0.76	-
14.	Terpineol	27.04	RI, MS	0.68	-
15.	β-Caryophyllene	38.01	RI, MS	**5.65**	-
16.	Isobornyl acetate	39.83	RI, MS	**28.01**	-
17.	α-Caryophyllene oxide	42.00	RI, MS	3.62	0.11
18.	Spathulenol	42.03	RI, MS	4.02	-
19.	Widdrol	43.01	RI, MS	0.81	-
20.	Nonanol	45.11	RI, MS	0.03	-
21.	Safranal	45.76	RI, MS	0.32	-
22.	Geranyl acetate	46.87	RI, MS	0.42	-
23.	2-Tridecenal	46.98	RI, MS	0.15	0.36
24.	Pyrrole	47.04	RI, MS	0.92	-
25.	2-Butanone	48.07	RI, MS	0.45	-
26.	Tricosane	48.15	RI, MS	0.43	-
27.	Tetracosane	49.00	RI, MS	2.31	-
28.	Pentacosane	49.85	RI, MS	2.54	0.19
29.	Hexacosane	49.87	RI, MS	0.32	-
30.	Heptacosane	49.95	RI, MS	0.19	1.81
31.	Dibutyl phthalate	50.01	RI, MS	0.41	0.77
32.	3-Hexanol	8.00	RI, MS	0.80	0.20
33.	2-Heptanol	8.33	RI, MS	0.76	0.29
34.	Orcinol	11.02	RI, MS	-	0.11
35.	Linalool Oxide	12.46	RI, MS	-	0.13
36.	Carvomenthone	14.44	RI, MS	-	0.09
37.	Acetophenone	15.74	RI, MS	-	0.20
38.	1,8-Menthadien-4-ol	15.97	RI, MS	-	0.08
39.	Cymene	16.16	RI, MS	-	0.17
40.	Δ-Terpineol	16.43	RI, MS	-	0.10
41.	Thymol	19.21	RI, MS	-	0.23
42.	β-Damascenone	23.14	RI, MS	-	0.13
43.	Indole	23.36	RI, MS	-	0.67
44.	Pentadecanol	26.26	RI, MS	-	0.53
45.	Lauric acid	28.74	RI, MS	-	0.28
46.	α-Benzenemethanol,4-Hydroxy	29.19	RI, MS	-	0.18
47.	Caryophyllene oxide	29.35	RI, MS	-	0.34
48.	4 H-Pyran-4-one, 2,6-dimethyl	29.98	RI, MS	-	0.13
49.	Octadecanol	30.13	RI, MS	-	0.14
50.	Heptanol	30.76	RI, MS	-	0.20
51.	Dimethylamphetamine	31.22	RI, MS	-	0.12
52.	N-Isopropyl-3-phenylpropanamide	31.29	RI, MS	-	0.20
53.	γ-Dehydro-ar-himachalene	31.38	RI, MS	-	0.80
54.	1,7-Octadiene	31.48	RI, MS	-	0.14
55.	Palmitic aldehyde	32.10	RI, MS	-	0.31
56.	Naphthalene	32.23	RI, MS	-	0.61
57.	Hexadecanol	32.61	RI, MS	-	0.59
58.	Myristyl aldehyde	32.90	RI, MS	-	0.14
59.	3-Tetradecene	33.03	RI, MS	-	0.33
60.	Cinnamaldehyde	33.30	RI, MS	-	0.50
61.	Valeric acid	33.67	RI, MS	-	1.69
62.	Menthyl lactate	34.04	RI, MS	-	0.43
63.	2-Methylundecanal	34.53	RI, MS	-	1.69
64.	Salicylate	34.59	RI, MS	-	1.50
65.	2-Bromoethanol	34.76	RI, MS	-	0.33
66.	β-Citronellol	34.92	RI, MS	-	0.59
67.	Decanol	35.20	RI, MS	-	0.58
68.	2-Pentadecanone	35.43	RI, MS	-	**8.65**
69.	Diethyl phthalate	35.79	RI, MS	-	2.47
70.	n-Hexadecanol	36.27	RI, MS	-	1.91
71.	2-Amino-5-(4-chlorophenyl)-1,3,4-thiadiazole	37.28	RI, MS	-	1.92
72.	Nonanoic acid	38.40	RI, MS	-	**34.41**
73.	3,7-Dimethyldibenzothiophene	39.19	RI, MS	-	2.44
74.	Octadecene	39.31	RI, MS	-	1.35
75.	Manool	41.00	RI, MS	-	0.45
76.	Verbanol	42.51	RI, MS	-	1.47
77.	I-Menthol	42.74	RI, MS	-	1.58
78.	Iridolactone	43.44	RI, MS	-	1.81
79.	Epilongipinanol	43.61	RI, MS	-	**5.19**
80.	Stearic acid	44.30	RI, MS	-	1.53
81.	Nonadecane	47.74	RI, MS	-	1.11
82.	Δ-Nonalactone	48.66	RI, MS	-	0.60
83.	Eicosane	51.79	RI, MS	-	3.07
84.	Docosane	54.02	RI, MS	-	0.73
**TOTAL**				**85.29**	**88.02**

The numbers highlighted in bold in the table represent the major components.

**Table 6:** The antimicrobial sensitivity of plants to disease-agent microorganisms using the agar disc diffusion method (the dose of plant extracts in 300 µL: 60 mg/µL).

Microorganisms	*M. laevis*	*M. spinosa*	Methanol	Standard Antibiotics
*E*. *coli*	14.33 ± 0.3 ^d^	-	-	19.33 ± 0.3 ^cd^*
*P. aeruginosa*	11.6 ± 0.3 ^d^	11.6 ± 0.3 ^d^	10.3 ± 0.3 ^d^	14.33 ± 0.3 ^d^*
*S*. *aureus*	12.3 ± 0.3 ^d^	10.66 ± 0.3 ^d^	12.6± 0.3 ^d^	19.33 ± 0.3 ^cd^*
*E*. *faecalis*	10.66 ± 0.3 ^d^	12.66 ± 0.3 ^d^	13.6± 0.3 ^d^	19.66 ± 0.3 ^cd^*
*C*. *albicans*	-	-	-	28.33 ±0.33 ^cd^**
*C*. *glabrata*	-	-	-	28.66 ± 0.33 ^cd^**
*C. tropicalis*	9.3 ± 0.3 ^d^	9.3 ± 0.3 ^d^	10.3 ± 0.3 ^d^	30.66 ± 0.33 ^cd^**
*E. floccosum*	28.3 ± 0.3 ^cd^	28.3 ± 0.3 ^cd^	-	19.66 ± 0.33 ^cd^**

Positive control: ampicillin–sulbactam (*) and nystatine (**) (10 µg disc^−1^ and 20 µg/disc). No negative control was used. Inhibition zone >15 mm (highly inhibitory effect; *p* < 0.0001; cd); 9–14 mm (effective; *p* < 0.001; d). Quantitative data expressed as mean ± standard error (mean ± SD).

**Table 7:** The inhibitory effect of plant extracts by minimum inhibition concentration (MIC in 500 μL—100 mg/μL).

Microorganism	MIC Values of *M. laevis*	MIC Values of *M. spinosa*	MIC Values of Methanol
*E. coli*	-	-	-
*P. aeruginosa*	62.5	62.5	62.5
*S. aureus*	-	62.5	62.5
*E*. *faecalis*	62.5	-	62.5
*C*. *albicans*	-	-	62.5
*C*. *glabrata*	-	-	62.5
*C. tropicalis*	-	-	125
*E. floccosum*	-	-	62.5

-: All four dilutions of the plant extract had no effect. The first dose was started 500 μL: 100 mg/μL.

**Table 8:** The DPPH% results of extracts of *Moluccella* L. taxa.

	100 mg/mL	250 mg/mL	500 mg/mL	750 mg/mL	1000 mg/mL
*M. laevis*	10.93 ± 0.45	27.23 ± 0.61	56.91 ± 0.06	81.47 ± 0.53	84.15 ± 0.59
*M. spinosa*	1.22 ± 0.09	16.74 ± 0.85	40.17 ± 1.79	51.56 ± 0.93	73.66 ± 1.06
Vitamin C	99.9 ± 0.2				
%99.9 MeOH	2.7 ± 0.3				

## Data Availability

Data are contained within the article.

## References

[1] Harley R.M., Atkins S., Budantsev A.L., Cantino P.D., Conn B.J., Grayer R., Harley M.M., De Kok R., Krestovskaja T., Morales R., Kubitzki K. (2004). Labiatae. The Families and Genera of Vascular Plants.

[2] Hamed A., Ahmed N., Attia E., Desoukey S. (2020). Phytochemical investigation of saponifiable matter & volatile oils and antibacterial activity of *Moluccella laevis* L., family Lamiaceae (Labiatae). J. Adv. Biomed. Pharm. Sci..

[3] Abdelaty N., Shihata E., Hamed A., Yehia S. (2021). Biological Potential of *Moluccella laevis* L. Aerial Parts, Family Lamiaceae (Labiatae). J. Adv. Biomed. Pharm. Sci..

[4] Doorandishan M., Pirhadi S., Swilam M.M., Gholami M., Ebrahimi P., El-Seedi H.R., Jassbi A.R. (2021). Molecular docking and simulation studies of a novel labdane type diterpene from *Moluccella aucheri* Scheen (Syn. Otostegia aucheri) as human-AChE inhibitör. J. Mol. Struct..

[5] Casiglia S., Jemia M.B., Riccobono L., Bruno M., Scandolera E., Senatore F. (2015). Chemical composition of the essential oil of *Moluccella spinosa* L. (Lamiaceae) collected wild in Sicily and its activity on microorganisms affecting historical textiles. Nat. Prod. Res..

[6] Jaradat N., Al-Masri M., Zaid A.N., Hussein F., Shadid K.A., Al-Rimawi F., Shayeb K., Sbeih A., Eid A. (2018). Assessment of the antimicrobial and free radical scavenging activities of *Moluccella spinosa*, *Helichrysum sanguineum*, and *Styrax officinalis* folkloric medicinal plants from Palestine. Orient. Pharm. Exp. Med..

[7] Demirpolat A. (2023). Essential Oil Composition Analysis, Antimicrobial Activities, and Biosystematic Studies on Six Species of *Salvia*. Life.

[8] Wodehouse R.P. (1935). Pollen Grains, Their Structure, Identification and Significancein Science and Medicine.

[9] Demirpolat A. (2022). Anatomical and Palynological Characteristics of Endemic *Fritillaria gencensis* Yıld., Kılıç & A. Demirpolat. Turk. J. Agric. Nat. Sci..

[10] Demirpolat A. (2022). Anatomical, Palynological and Seed Surface Characteristics of *Aethionema sancakense* Yıld. & Kılıc (Brassicaceae). Eur. J. Sci. Technol..

[11] Faegri K., Iversen J. (1954). Textbook of Modern Pollen Analysis. Weather.

[12] Demirpolat A. (2022). Chemical Composition of Essential Oils of Seven *Polygonum* Species from Turkey: A Chemotaxonomic Approach. Molecules.

[13] Gupta C., Garg A.P., Uniyal R.C., Kumari A. (2008). Comparative Analysis of The Antimicrobial Activity of *Cinnamon* Oil and *Cinnamon* Extract on somefood-borne microbes. Afr. J. Microbiol. Res..

[14] Torğut G., Pıhtılı G., Erecevit Sönmez P., Erden Y., Kırbağ S. (2020). Synthesis and Antimicrobial and Anticancer Activities of Sodium Acrylate Copolymers. J. Bioact. Compat. Polym..

[15] NCCLS (2000). Methods for Dilution and Antimicrobial Susceptibility Tests for Bacteria That Grow Aerobically.

[16] Liyana-Pathiranan C.M., Shahidi F. (2005). Antioxidant activity of commercial soft and hard wheat (*Triticum aestivum* L.) as affected by gastric pH conditions. J. Agric. Food Chem..

[17] Yılmaz Sancar P., İnci Ş., Demirpolat A., Kırbağ S., Civelek Ş. (2024). Antimicrobial, antioxidant and essential oil studies on *Veratrum album* L. (Melanthiaceae). Int. J. Second. Metab..

[18] Meng Z.L., Qin R.X., Wen R.S., Li G.Q., Liang Z.Y., Xie J.K., Zhou Y.H., Yang Z.Q. (2023). Study on Synthesizing Isobornyl Acetate/Isoborneol from Camphene Using α-Hydroxyl Carboxylic Acid Composite Catalyst. Molecules.

[19] Ventola C.L. (2015). The antibiotic resistance crisis: Part 1: Causes and threats. Pharm. Ther..

[20] Davis P.H. (1982). Flora of Turkey and the East Aegean Islands.

[21] Abdelaty N.A., Attia E.Z., Hamed A., Desoukey S.Y. (2019). Morphoanatomy studies of the leaves of of *Moluccella laevis* L. Family: Lamiaceae (Labiatae), cultivated in Egypt. J. Pharm. Phytochem..

[22] Shehata I. (2001). A pharmacognostical study of *Moluccella laevis* L.. Bull. Fac. Pharm. Cairo Univ..

[23] Bakhshi O., Bagherzade G., Ghamari Kargar P. (2021). Biosynthesis of Organic Nanocomposite Using *Pistacia vera* L. Hull: An Efficient Antimicrobial Agent. Bioinorg. Chem. Appl..

[24] Tschugaeff L. (1900). Ueber das Thujen, ein neues bicyclisches Terpen. Berichte Dtsch. Chem. Ges..

[25] Nikitina L.E., Startseva V.A., Vakulenko I.A., Khismatulina I.M., Lisovskaya S.A., Glushko N.P., Fassakhov R.S. (2009). Synthesis and antifungal activity of compounds of the pinane series. Pharm. Chem. J..

[26] Silva A.C.R., Lopes P.M., Azevedo M.M.B., Costa D.C.M., Alviano C.S., Alviano D.S. (2012). Biological activities of α-Pinene and β-Pinene enantiomers. Molecules.

[27] Felipe C.F.B., Albuquerque A.M.S., de Pontes J.L.X., de Melo J.V., Rodrigues T.C.M.L., de Sousa A.M.P., Monteiro A.B., da Silva Ribeiro A.E., Lopes J.P., Menezes I.R.A. (2019). Comparative study of α and β-Pinene effect on PTZ-induced convulsions in mice. Fund. Clin. Pharmacol..

[28] Salehi B., Upadhyay S., Orhan I.E., Jugran A.K., Jayaweera S.L.D., Dias D.A., Sharopov F., Taheri Y., Martins N., Baghalpour N. (2019). Therapeutic Potential of α- and β-Pinene: A Miracle Gift of Nature. Biomolecules.

[29] Anneken D.J., Both S., Christoph R., Fieg G., Steinberner U., Westfechtel A. (2006). Fatty Acids. Ullmann’s Encyclopedia of Industrial Chemistry.

[30] Chang P., Terbach N., Plant N., Chen P.E., Walker M.C., Williams R.S. (2013). Seizure control by ketogenic diet-associated medium chain fatty acids. Neuropharmacology.

[31] Aneja M., Gianfagna T.J., Hebbar P.K. (2005). *Trichoderma harzianum* produces nonanoic acid, an inhibitor of spore germination and mycelial growth of two cacao pathogens. Physiol. Mol. Plant Pathol..

[32] Miller T.W. (2006). Natural herbicides and amendments for organic weed control. Crop Protection Products for Organic Agriculture.

[33] Yannai S. (2003). Dictionary of Food Compounds with CD-ROM: Additives, Flavors, and Ingredients.

[34] Desai A.G., Qazi G.N., Ganju R.K., El-Tamer M., Singh J., Saxena A.K., Bedi Y.S., Taneja S.C., Bhat H.K. (2008). Medicinal plants and cancer chemoprevention. Curr. Drug Met..

[35] Faydaoğlu E., Sürücüoğlu M.S. (2011). History of the Use of Medical and Aromatic Plants and their Economic Importance. Kastamonu Üniversitesi Orman Fakültesi Derg..

[36] Dar R.A., Shahnawaz M., Qazi P.H. (2017). General overview of medicinal plants: A review. J. Phytophar..

[37] Rawat D., Shrivastava S., Naik R.A., Chhonker S.K., Mehrotra A., Koiri R.K. (2018). An overview of natural plant products in the treatment of hepatocellular carcinoma. Anti-Cancer Agents Med. Chem..

[38] Ginwala R., Bhavsar R., Chigbu D.I., Jain P., Khan Z.K. (2019). Potential role of flavonoids in treating chronic inflammatory diseases with a special focus on the anti-inflammatory activity of apigenin. Antioxidants.

